# Acriflavine: an efficient green fluorescent probe for sensitive analysis of aceclofenac in pharmaceutical formulations

**DOI:** 10.1186/s13065-023-00979-2

**Published:** 2023-08-02

**Authors:** Amal A. El-Masry, Abdallah M. Zeid

**Affiliations:** 1 Department of Medicinal Chemistry, Faculty of Pharmacy, Mansoura University, Mansoura, 35516 Egypt; 2 Department of Pharmaceutical Analytical Chemistry, Faculty of Pharmacy, Mansoura University, Mansoura, 35516 Egypt

**Keywords:** Acriflavine, Aceclofenac, Fluorescent probe, Stern–Volmer plot, Dosage form

## Abstract

Acriflavine is a multipurpose drug that shows antibacterial, antiviral, antimalarial, and antifungal activities. The remarkable native fluorescence of acriflavine is exploited in analytical chemistry field as an efficient probe for analysis of pharmaceutical and biological compounds. The fluorescent probe action of acriflavine is based on the remarkable fluorescence turning-off via formation of ion-pair complexes with acidic drugs at a specific pH. Herein, the acidic drug aceclofenac is analysed for the first time using acriflavine as a fluorescent probe. Aceclofenac can form an ion-pair complex with acriflavine at pH 8.5, and hence it partially turns off the fluorescence intensity of acriflavine over a concentration range of 1–20 µg/mL. The fluorescence quenching was monitored at 502 nm following an excitation at 265 or 451 nm. The reaction stoichiometry between acriflavine and aceclofenac was found to be 1:1 using limiting logarithmic method. The type of quenching was confirmed to be static using Stern–Volmer plot. The method showed low values of quantitation limit (0.89 µg/mL) and detection limit (0.29 µg/mL). Moreover, the method was linear (r = 0.9999), accurate, precise (RSD < 1.7%), robust, and specific. The proposed method was successfully employed to analyse aceclofenac in its dosage forms with high %recovery (98–101%). Additionally, GAPI and AGREE approaches were used to guarantee the suggested techniques' greenness, and the findings showed an excellent level of greenness.

**Supplementary Information:**

The online version contains supplementary material available at 10.1186/s13065-023-00979-2.

## Introduction

**Fig. 1:**
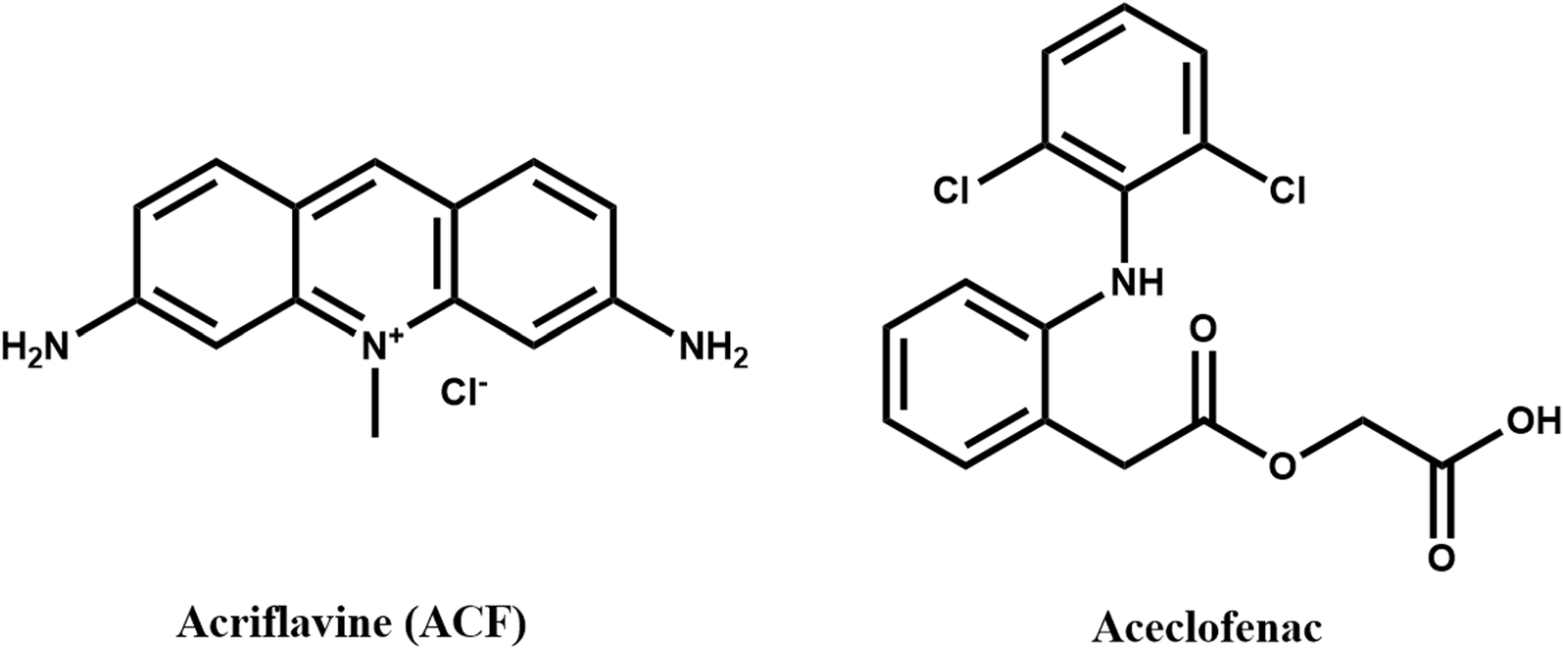
Chemical structures of aceclofenac drug and acriflavine (ACF) fluorescent probe

Acriflavine (ACF) (Fig. 1) is a multipurpose drug that was used as an antibacterial drug before the discovery of penicillin [1, 2]. Besides its antibacterial activity, ACF is reported to have antiviral (against HIV), antimalarial, and anticancer activities [3–5]. Recently, it was proved that ACF has a powerful inhibitory effect on SARS-CoV-2 and hence it can be used for treatment of COVID-19 infection [1, 6]. Besides its biological and medicinal functions, it can be used as a selective fluorescent probe for analysis of acidic compounds such as ascorbic acid [7], sulfasalazine [8], and cefipime [9]. Its analytical applications are mainly based on its native fluorescence properties that could be exploited to be used as a fluorescent probe for analysis of various compounds. As a fluorescent probe, it can be quenched via the interaction of its basic function groups with acidic compounds (carboxylate or sulfonates), forming ion-pair complexes with lower fluorescent properties [7, 9, 10]. Therefore, its application could be extended to numerous acidic pharmaceutical compounds. In our study, aceclofenac was selected as a model example to study the interaction between ACF and acidic compounds for selective quantitative analysis of such compounds.

Aceclofenac, (2-[2-[2-[(2,6-dichlorophenyl)amino]phenyl]acetyl]oxyacetic acid, Fig. 1) is a mono carboxylic acid drug belonging to non-steroidal anti-inflammatory drugs (NSAID) [11]. It is widely used to relieve the painful inflammations associated with low back pain, ankylosing spondylitis, extraarticular rheumatism, scapulohumeral periarthritis, rheumatoid arthritis, and osteoarthritis [11, 12].

Numerous methods were reported for the analysis of aceclofenac in pharmaceutical formulations and/or biological fluids. The reported methods include spectrophotometric [13–16], spectrofluorometric [15], liquid chromatographic [17–20], capillary electrophoretic [21], and electrochemical [22, 23] methods. The spectrofluorimetric method [15] relied on monitoring the native fluorescence of aceclofenac in a phosphate buffer medium (pH 8) at 355 nm after excitation at 250 nm. The reported method had a narrow linear range of 2–8 µg/mL. Therefore, we aimed to develop a more sensitive method with a wider linear range for analysis of aceclofenac.

Herein, ACF fluorescent probe was used for the first time to analyse aceclofenac with a wider linear range (1–20 µg/mL) and a low detection limit (0.29 µg/mL). The merits of the proposed method involved simplicity, specificity, high accuracy, adequate sensitivity, and rapidity. In addition, the evolved method was employed to analyse aceclofenac in its pharmaceutical dosage form with low RSD (< 1.5%) and high recovery values (98–101%). The greenness of the developed method was estimated using Green Analytical Procedure Index (GAPI) and Analytical GREEnness (AGREE) approaches, which confirmed the excellent greenness of the fluorescent probe.

## Experimental

### Instrumentation

Fluorescent measurements of ACF before and after its interaction with aceclofenac target analyte were performed using Cary Eclipse fluorescence spectrophotometer. The spectrofluorimetric instrument is equipped with an Agilent Xenon flash lamp. The fluorescence quenching measurements of ACF sensor were monitored at 502 nm upon excitation at 265 or 451 nm. A smoothing factor of 20 was used for all measurements. The pH adjustments were performed using a Consort pH-meter (NV P-901, Belgium). The vortex mixer (IVM-300p, Taiwan) was used for homogenous mixing the solutions.

The comparison UV spectrophotometric method was performed by direct analysis of aceclofenac at 203 nm on the recorded zero order spectrum using methanol: water (50: 50, v/v) as the optimum solvent.

### Materials and reagents

Aceclofenac (purity: 98.7%) was procured from SmithKline Beecham, (Egypt). Acriflavine (99.5% purity) was purchased from Eva Pharma Company (Cairo, Egypt). Bristaflam^®^ tablets (Batch number: 62000000056773), labeled to contain 100 mg of aceclofenac in each enteric coated tablet, was procured from local pharmacies, Egypt.

Boric acid and sodium hydroxide were acquired from Piochem Co, Egypt. Orthophosphoric acid and glacial acetic acid (99%), were purchased from EL-Nasr pharmaceutical company (ADWIC).

Britton Robinson buffer (BRB) solutions of pH ranging from 2.0 to 12.0 were prepared via mixing equal concentrations of acetic acid, boric acid, and phosphoric acid (40 mM each) and adjustment of the solutions’ pH using 0.2 M NaOH.

### Standards

Aceclofenac standard solution (100 μg mL^−1^) was prepared via dissolving 10.0 mg aceclofenac raw material in 100.0 mL ethanol. The acriflavine stock solution of 0.8 mM was performed via dissolution of 20.8 mg ACF in 100.0 mL distilled water. The prepared stock solution was further diluted to 8 × 10^−3^ mM by transferring 1.0 mL of ACF stock solution (0.8 mM) into a 100.0 mL volumetric flask and completing it to 100.0 mL with distilled water.

The working solution of ACF (8 × 10^−4^ mM) was daily prepared in dist. water to be employed for fluorescent measurements of aceclofenac samples.

### Analysis of standards

**Fig. 2:**
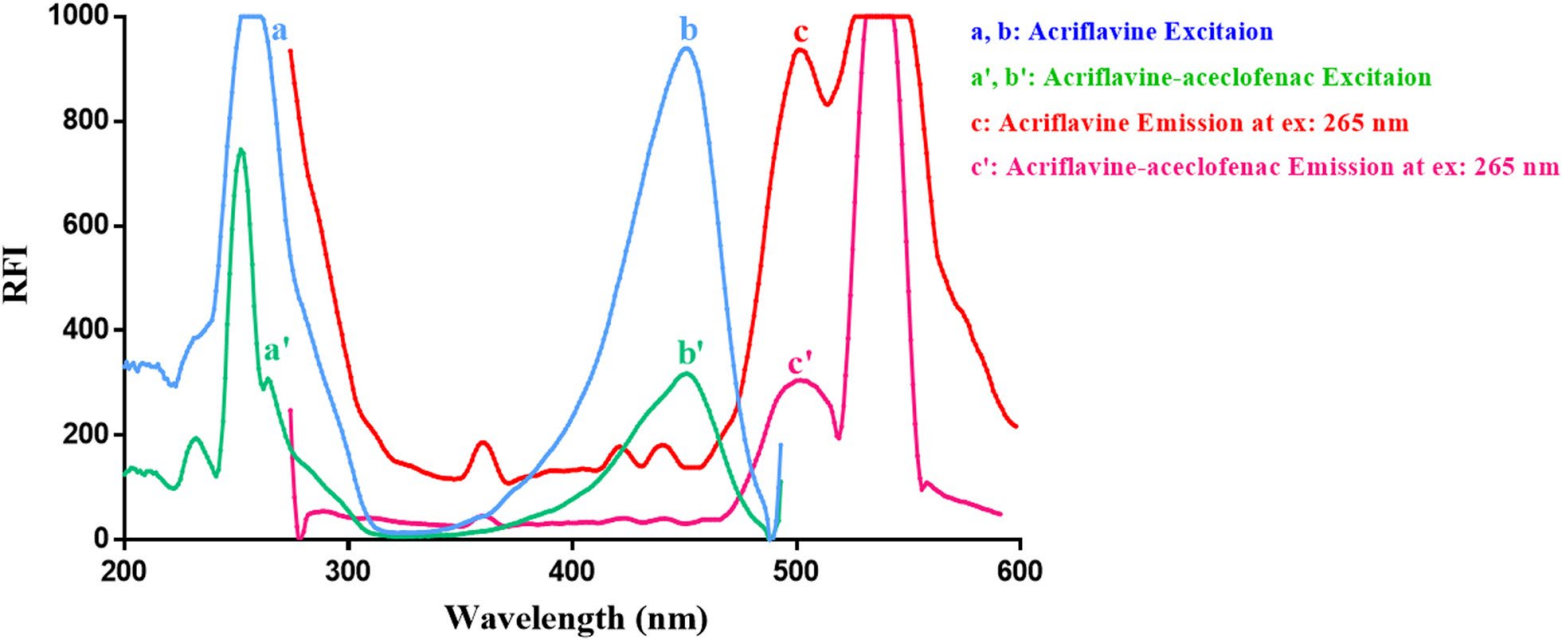
Fluorescent excitation (**a**, **b**) and emission (**c**) spectra of 8 × 10^−7^ M acriflavine (ACF) alone, and excitation (**a′**, **b′**) and emission (**c′**) spectra of 8 × 10^−7^ M ACF after reaction with 20 μg ml^−1^ aceclofenac upon adjusting the emission wavelength at 502 nm and the excitation wavelength at 265 nm

**Fig. 3:**
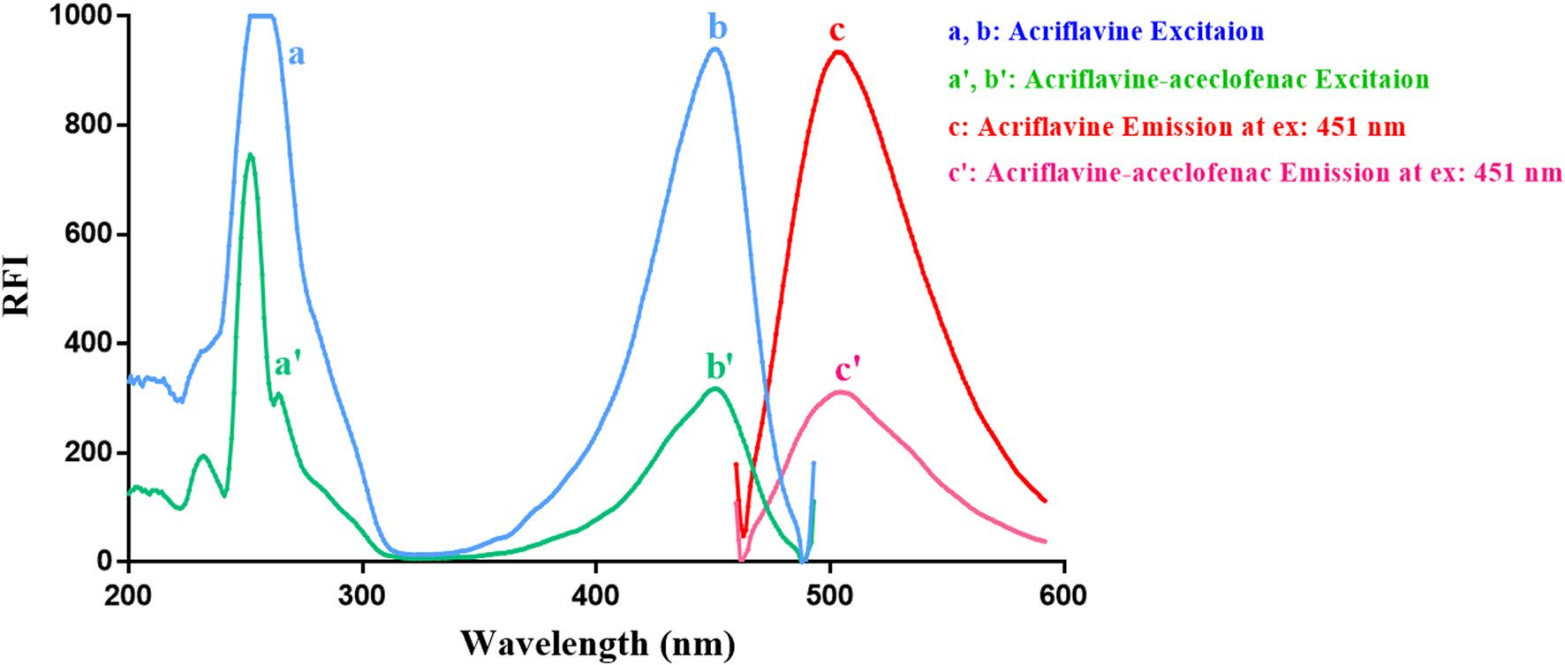
Fluorescent excitation (**a**, **b**) and emission (**c**) spectra of 8 × 10^−7^ M acriflavine (ACF) alone, and excitation (**a′**, **b′**) and emission (**c′**) spectra of 8 × 10^−7^ M ACF after reaction with 20 μg ml^−1^ aceclofenac upon adjusting the emission wavelength at 502 nm and the excitation wavelength at 451 nm

Different quantities of aceclofenac within the concentration range (1.0–20.0 μg mL^−1^) were placed into a series of 10-mL measuring flasks. Thereafter, one milliliter of the buffer (BRB solution adjusted at pH 8.5) and one milliliter of ACF solution (8 × 10^−4^ mM) were added, and the flasks were completed to the mark utilizing water, mixed well, and left for at least 2 min before fluorescence analysis. The remarkable decrease in the fluorescence intensity of ACF was then monitored at 502 nm (λ_ex_ = 265 or 451 nm), as shown in Figs. 2 and 3. All measurements were performed against blank for accurate analysis. The calibration curve was built by drawing the fluorescence intensity decrease (Fluorescent intensity of blank − fluorescent intensity of aceclofenac-ACF ion − pair complex) with respect to the final drug concentrations in μg mL^−1^. Thereafter, the regression analysis was carried out.

### Assay of aceclofenac in tablets

Ten Bristaflam^®^ tablets were weighed, completely crunched for quantitative determination of aceclofenac. A specified weight (equivalent to 10.0 mg aceclofenac) of the crunched tablets was accurately placed into 100-mL measuring flask. Thereafter, 70 mL of ethanol were added, and the flask was sonicated for 20 min before completing the flask to 100.0 mL with ethanol. The content of the flask was then filtered via a 0.45 μm syringe filter; the filtrate has been diluted with ethanol, and the procedure outlined in the “Analysis of standards” section was followed. Calculation of nominal content in tablets has been calculated from regression equation.

### Estimation of quantum yield

The quantum yield of the effectively used ACF probe was estimated using the equation [24]:

$$\Phi_{{\text{x}}} = \, \Phi_{{{\text{st}}}} \times \left( {{\text{F}}_{{\text{x}}} /{\text{F}}_{{{\text{st}}}} } \right) \times \left( {\upeta_{{\text{x}}}/\upeta_{{{\text{st}}}} } \right)^{{2}} \times \left( {{\text{A}}_{{{\text{st}}}} /{\text{A}}_{{\text{x}}} } \right)$$

where, in that order, the absorbance, the solvent refractive index, the integrated intensity of emission, and quantum yield were denoted as (A, F, η and Φ); η_x_/η_st_ in aqueous solutions is equivalent to one. The subscript (x) denotes the unknown, whereas the subscript (st) denotes the reference fluorescein standard.

The reference fluorescein standard is prepared in 0.1 M NaOH, where its quantum yield is 0.93 [25]. The quantum yield of ACF was calculated using the above equation and its value was found to be 0.54 ± 0.03.

## Results and discussion

### Stepwise experimental optimisation

Turning off the remarkable fluorescence of ACF may be affected by various factors, which could sequentially be affected by the formed complex [aceclofenac-ACF; ion-association complex]. All parameters that could affect the formation of the complex, its stability or even the sensitivity of the method were studied to utilize the optimum parameters in each case.

#### Buffer pH and volume

**Fig. 4:**
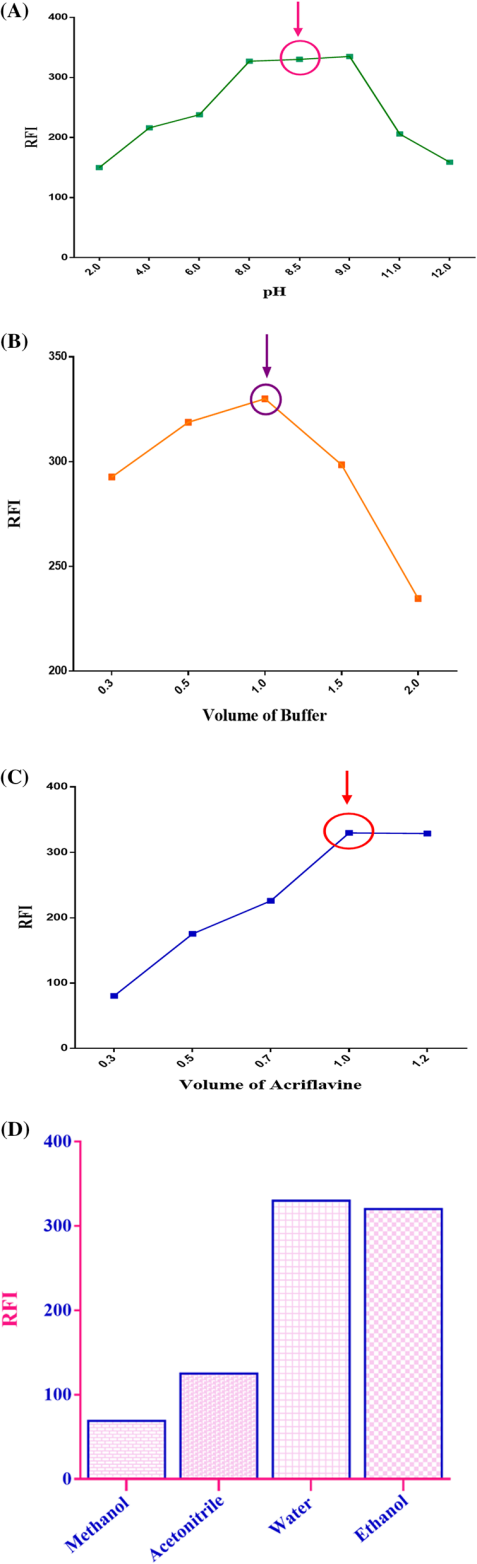
The influence of stepwise experimental parameters variation on the quantification of aceclofenac (10.0 µg/mL) adopting fluorescence of 8 × 10^−7^ M acriflavine (ACF); where **A** pH of buffer (BRB), **B** volume of buffer, **C** volume of ACF (8 × 10^−7^ M), and **D** diluting solvents

The analysis of aceclofenac (10.0 µg/mL) using ACF probe was carried out at a pH range between 2.0 and 12.0 using the BRB buffer. The highest ΔF values were observed at a pH ranging from 8.0 to 9.0; hence, the pH 8.5 was selected as the optimum pH for this work, as illustrated in Fig. 4A, as it has a significant influence on the formation of a stable ion-pair complex. At pH values greater than 9.0, a significant decrease in the fluorescence intensity was observed. To study the effect of buffer volume, different quantities of the BRB solution (pH 8.5) ranging from 0.3 to 2.0 mL were studied, and it was found that highest Δ*F* are obtained when utilizing only 1.0 mL, as shown in Fig. 4B.

#### Acriflavine volume

Different volumes of ACF (0.3–1.2 mL) were utilized to study the effect of ACF volumes on RFI. The maximum values of RFI were obtained upon using one milliliter as demonstrated in Fig. 4C.

#### Reaction medium

The effect of reaction medium was studied by investigating different solvents such as water, ethanol, methanol, and acetonitrile. Both ethanol and water exhibited high RFI upon analysis of aceclofenac, as demonstrated in Fig. 4D. On the other hand, both acetonitrile and methanol produced lower RFI values. Finally, we selected water as the solvent of choice because it rendered a relatively higher RFI values than ethanol and it is cheap, available, and safe.

### Explanatory mechanism of the reaction between aceclofenac and ACF

#### Stoichiometry of the reaction between aceclofenac and ACF

**Fig. 5:**
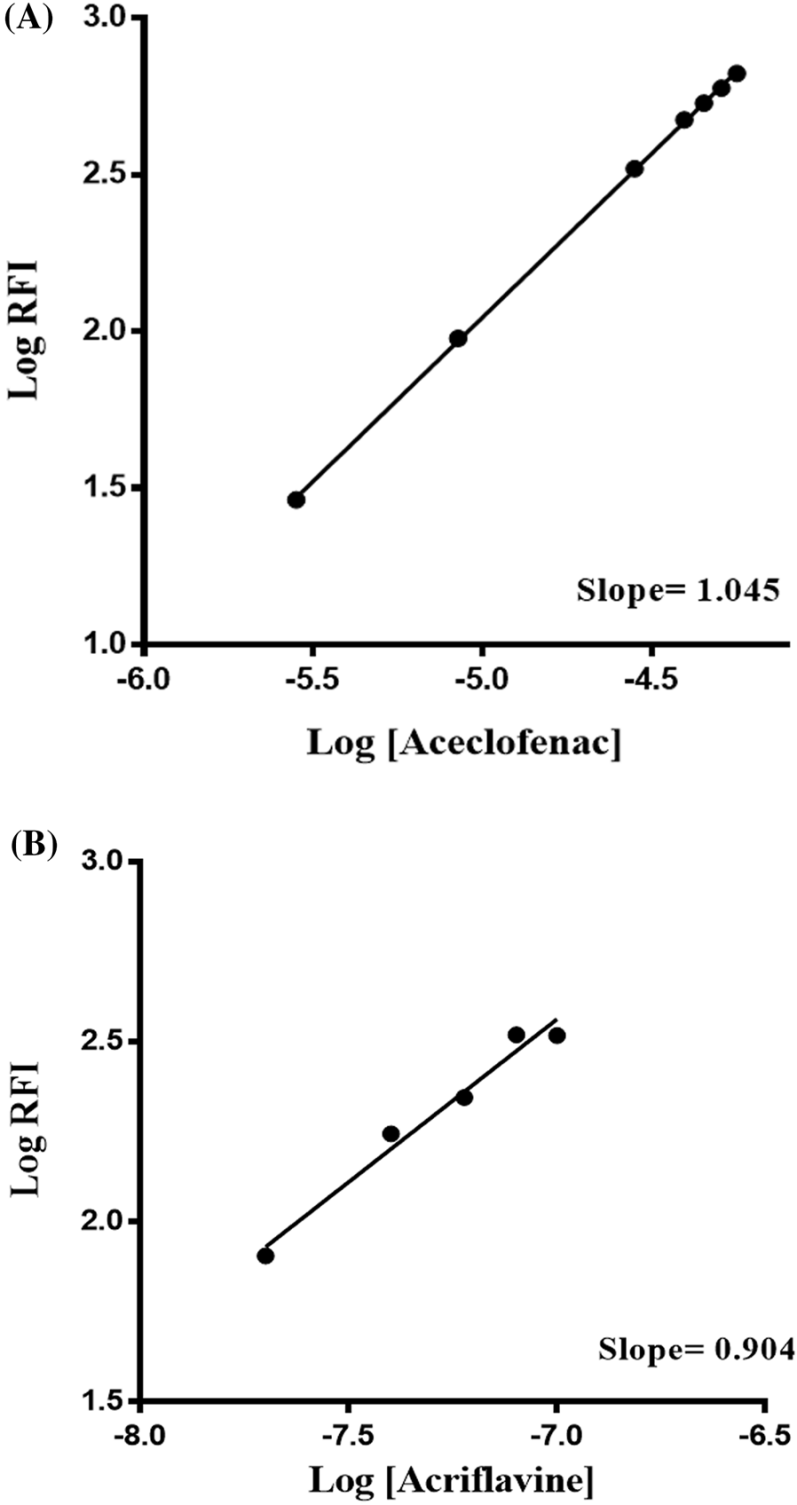
Stoichiometry of the fluorimetric interaction between aceclofenac and acriflavine (ACF) using limiting logarithmic method where: **A** log [aceclofenac] against log RFI, **B** log [ACF] against log RFI

**Fig. 6:**
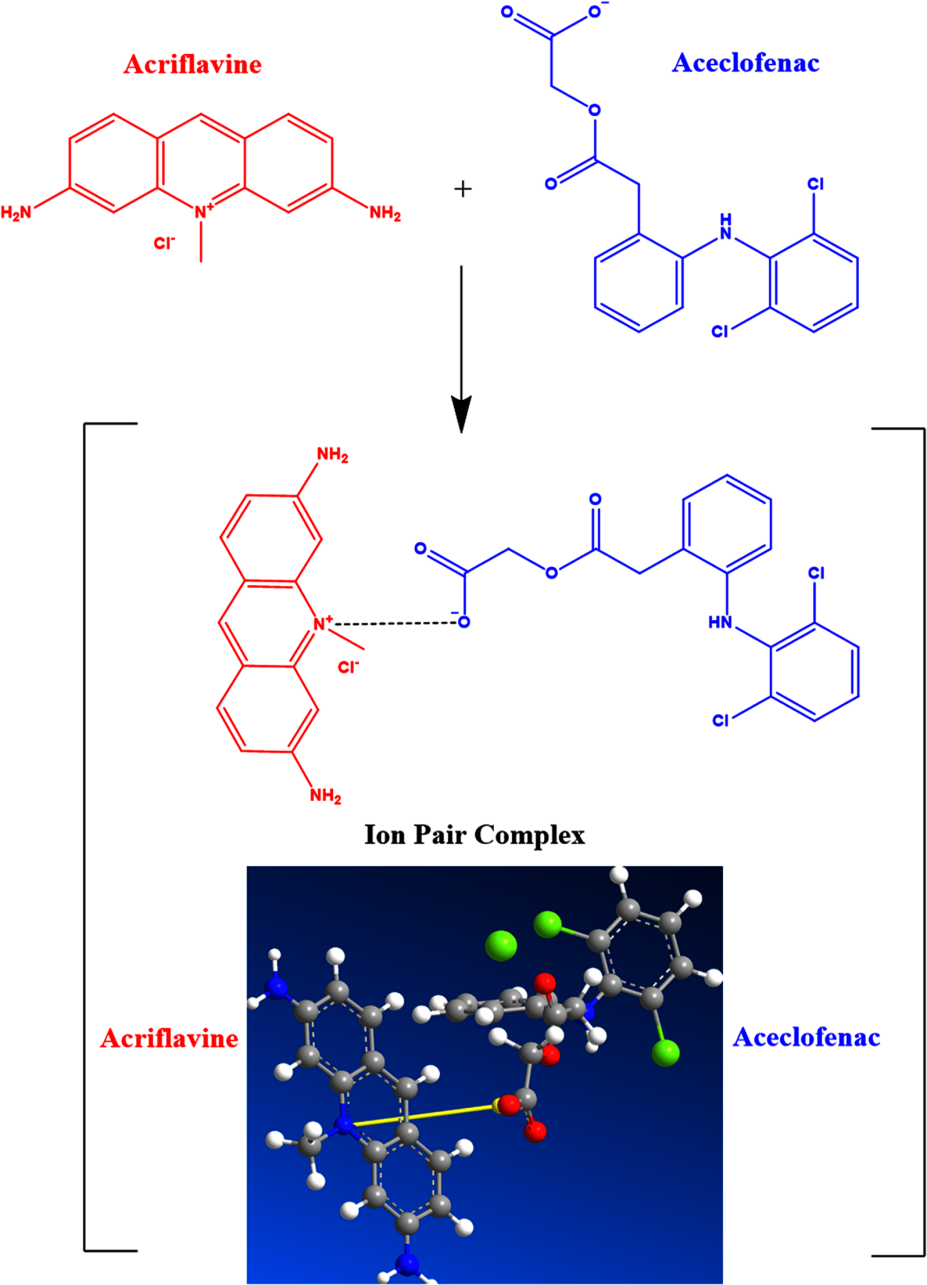
Schematic illustration of the reaction pathway between aceclofenac drug and aciflavine (ACF) to form the ion-associated complex

The alternate measurement of the Δ*F* of the reaction product at increasing conc. of either aceclofenac or ACF was done using a limiting logarithmic approach. As shown in Fig. 5, the log (RFI) *vs*. log [ACF] and log (RFI) *vs*. log [aceclofenac] plots both showed straight lines with slope values of 1.045 and 0.904, respectively. It was determined that the reaction's molar reactivity was 1.045/0.904, meaning that the ratio of ACF to aceclofenac was 1:1. The ratio may be calculated owing to aceclofenac's single carboxylic group. Aceclofenac's negatively charged carboxylate group and the ACF's positively charged nitrogen atom interacted at a particular pH. So, the electrostatic forces participated in the creation of an ion-association complex as illustrated in Fig. 6.

#### Mechanism of turning off the fluorescence of fluorescent probe (ACF) in response to aceclofenac

Turning off the fluorescence of ACF is mainly due to formation of ion-pair complex with acidic compounds (Aceclofenac). The cause of quenching may rely on the following possible interactions: collisional quenching, excited-state interreactions, molecular rearrangement, and ground-state complex formations. It is an important issue to assess the quenching type in our study to inspire the mechanism of quenching. Stern–Volmer curves are built up using the equation:

$${\text{F}}_{{\text{o}}} /{\text{F}}\, = \,{1}\, + \,{\text{K}}_{{{\text{SV}}}} [{\text{C}}].$$

where [C] is the drug's molar concentration, F and F_o_ are ACF’s relative fluorescent intensities when the drug is present and absent, respectively. K_SV_ stands as the Stern–Volmer constant. All results of the proposed method were conducted after adjusting excitation wavelength at 451 nm.

**Fig. 7:**
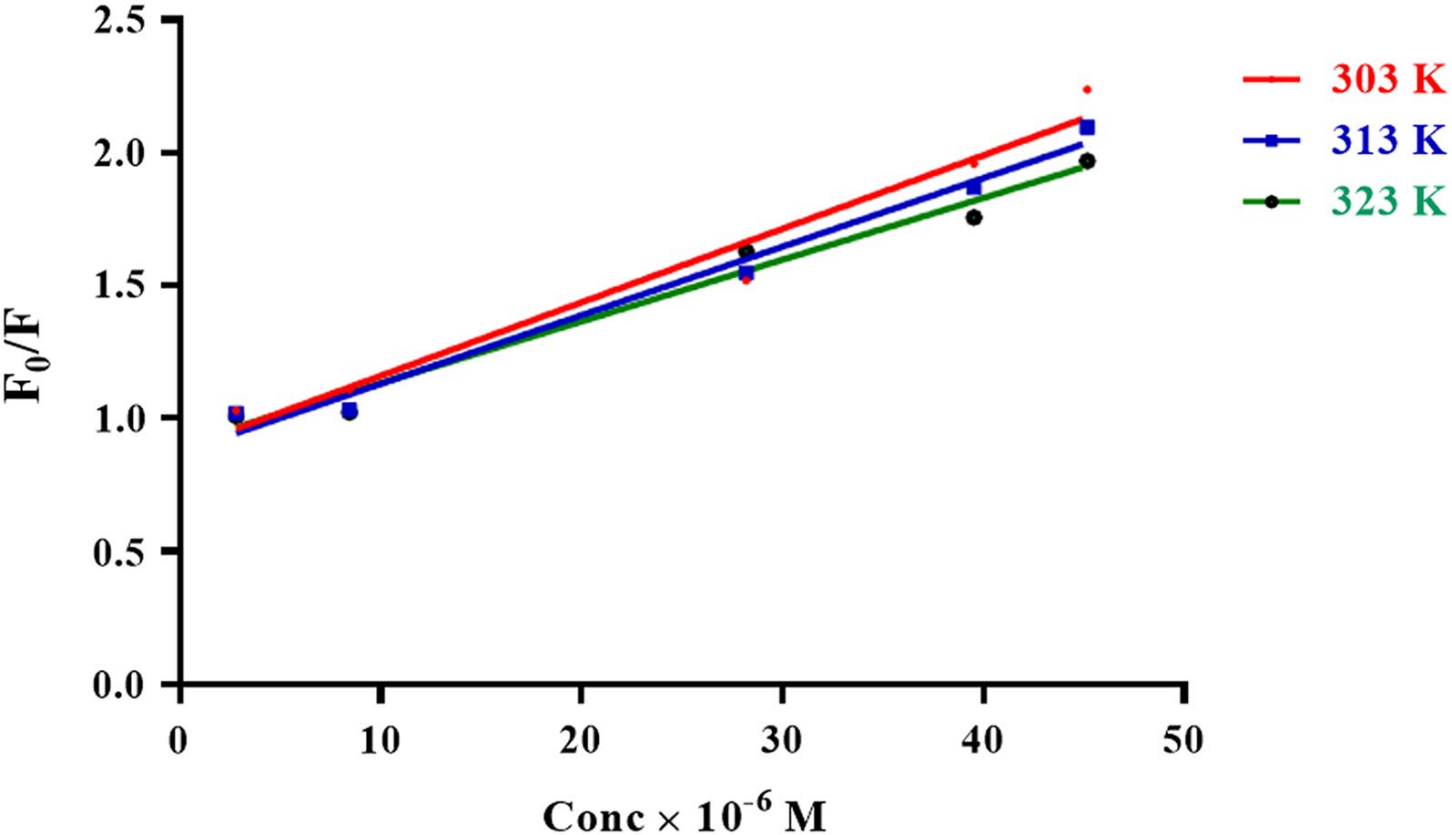
Stern–Volmer graphs for aceclofenac at 303, 313, and 323 K

By graphing F_o_/F against [C], the Stern–Volmer plots were created, and the linearity of the plots (r = 0.993, 0.992, and 0.99) predicts whether dynamic or static quenching will occur. Also, by creating Stern–Volmer graphs at three different temperatures, the temperature dependency was examined (Fig. 7).

The K_SV_ in Table 1 decreases as temperature rises, which is a sign of static quenching. We ultimately decided that ACF and aceclofenac create a non-fluorescent complex, and its stability is decreased upon increasing temperatures. The following equation was used to compute the bimolecular quenching constants (K_q_), which represent the fluorescence efficiency:

$${\text{K}}_{{\text{q}}} = {\text{ K}}_{{{\text{SV}}}} /{\text{t}}_{0} ,$$

where the luminescence lifetime of ACF is 5 × 10^−9^ s, so *K*_q_ was found to be 4.6–5.6 × 10^12^ l mol^−1^ s^−1^.

**Table 1:** Stern–Volmer parameters for the complex-formation reaction between aceclofenac and the fluorescent probe ACF

Temperature	30 °C (303 K)	40 °C (313 K)	50 °C (323 K)
Correlation coefficient (*r*)	0.993	0.992	0.990
Stern–Volmer quenching constant *K*_sv_ (l mol^−1^) K_SV_ = slope	0.028 × 10^6^	0.026 × 10^6^	0.023 × 10^6^
T_0_ (florescence lifetime)	5 × 10^–9^	5 × 10^–9^	5 × 10^–9^
Bimolecular quenching constant Kq = K_SV_/t_0_	5.6 × 10^12^	5.2 × 10^12^	4.6 × 10^12^

**Fig. 8:**
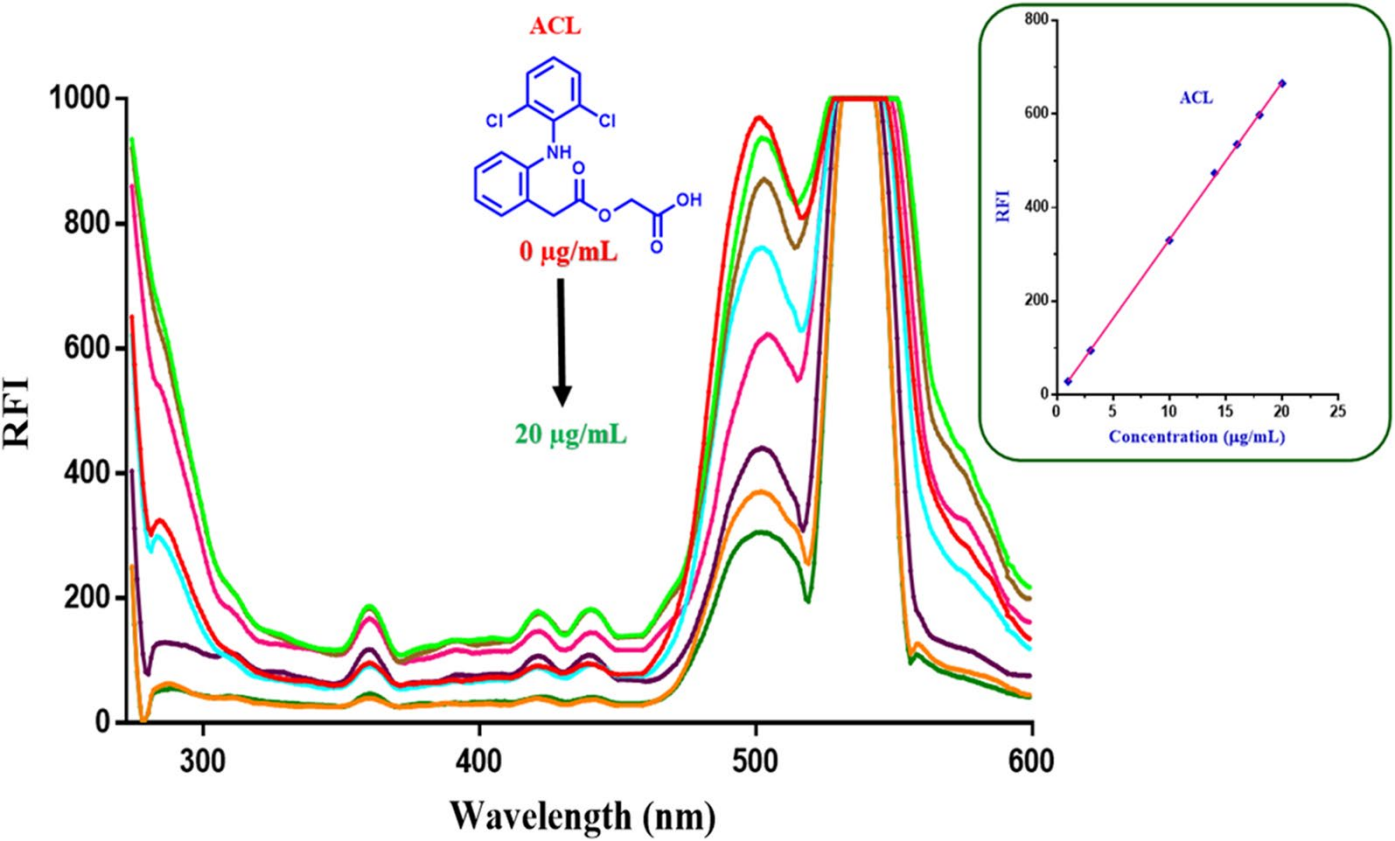
Quenching of the fluorescence emission of ACF in quantitative manner upon addition of increasing concentrations of aceclofenac (ACL) after excitation at 265 nm. From red to green: 0.0, 1.0, 3.0, 10.0, 14.0, 16.0, 18.0, 20.0 µg/mL of aceclofenac

**Fig. 9:**
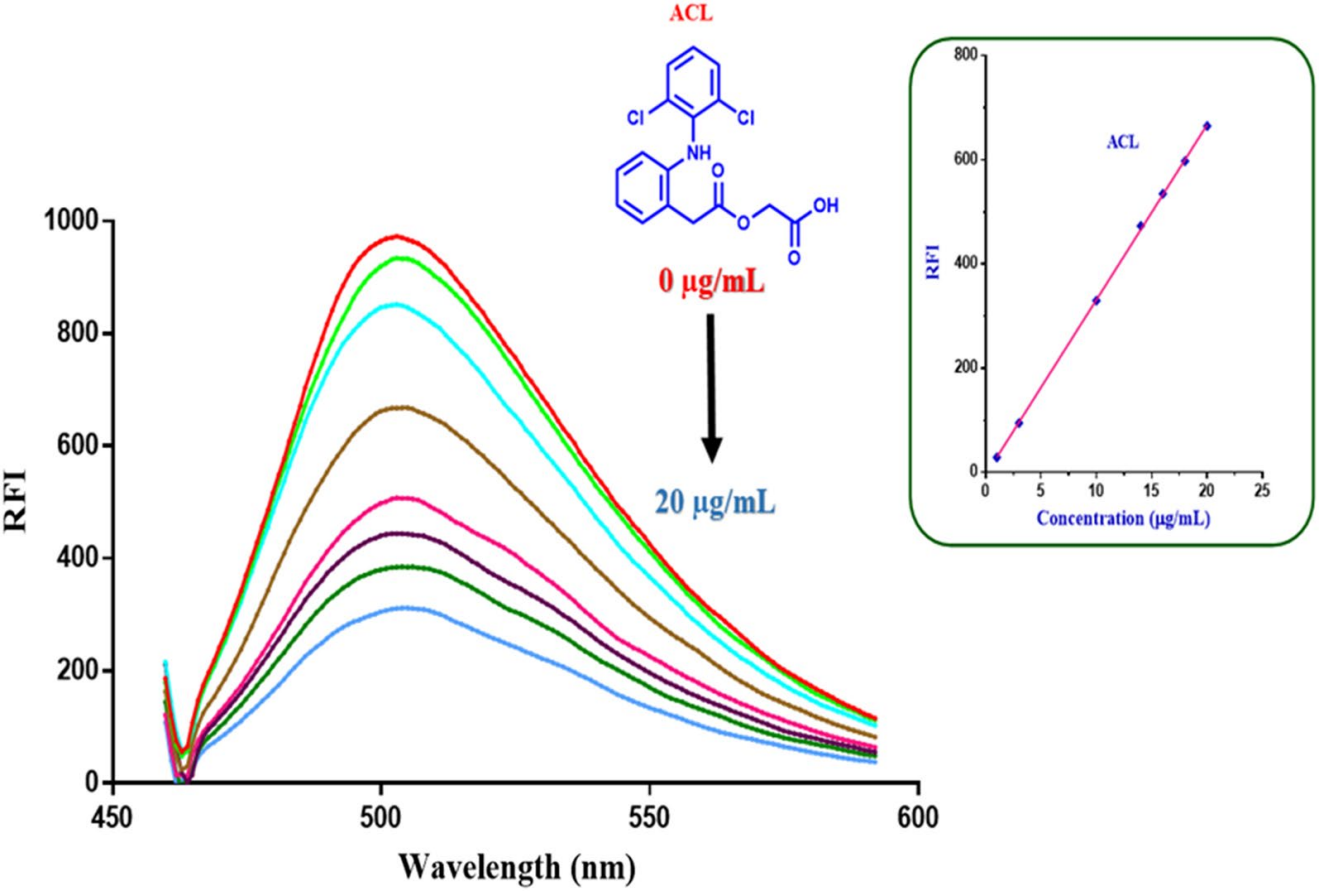
Turning-off the fluorescence emission of Acriflavine in quantitative manner upon addition of increasing concentrations of aceclofenac (ACL) after excitation at 451 nm. From red to blue (0.0, 1.0, 3.0, 10.0, 14.0, 16.0, 18.0, 20.0 µg/mL aceclofenac)

Figures 8 and 9 show the gradual decrease in the fluorescence intensity of ACF probe upon addition of increasing conc. of aceclofenac after excitation at 265 and 451 nm, respectively. This linear decrease in fluorescence intensity of ACF was employed for efficient quantitative assay of aceclofenac in raw material and pharmaceutical tablets.

### Validation

The evolved fluorescent method was fully validated according to the ICH Q2 (R1) guidelines [26]. All validation measurements were performed after adjusting the excitation wavelength at 451 nm.

The linearity was confirmed by the high value of correlation coefficient (r = 0.9999) for aceclofenac when the RFI was plotted against aceclofenac concentration. The method was rectilinear over the range of 1.0–20 µg/mL for aceclofenac as demonstrated in Table 2.

**Table 2:** Analytical data for the spectrofluorometric assay of aceclofenac using the acriflavine (ACF) as a fluorescent probe

Parameter	Range/value
Excitation and emission wavelength (λ_ex/em_)	451/502 nm
Concentration range	1.0–20.0 µg/mL
Correlation coefficient	0.9999
Intercept ± S_a_	− 4.39 ± 2.99
Slope ± S_b_	33.61 ± 0.22
Standard deviation of residuals	3.98
Limit of detection	0.29 µg/mL
Limit of quantification	0.89 µg/mL
% Relative standard deviation	0.93
%Error	0.35

S_a_: standard deviation of intercept; S_b:_ standard deviation of slope

Both detection and quantitation limits were calculated according to the ICH Q2 (R1) recommendations [26] using the following equations, LOD = 3.3 *S*_*a*_/*b* and LOQ = 10 *S*_*a*_*/b*, in which LOD refers to the limit of detection, LOQ refers to the limit of quantitation, "S_a_" denotes standard deviation of the intercept, and "b" denotes the slope of the calibration curve.

The results in Table 2 indicates low values of LOD (0.29 µg/mL) and LOQ (0.89 µg/mL), which are suitable to assay aceclofenac in its pharmaceutical dosage form.

To assess the accuracy of the developed method, a comparison spectrophotometric method [16] was performed to compare the results with those obtained from our proposed method. The obtained results (Table 3) demonstrated that no significant difference between the proposed and the comparison methods by applying *t-* and F-tests [27].

**Table 3:** Comparison between the proposed spectrofluorometric method and the reported spectrophotometric one relying on the % recoveries of aceclofenac in pure form

Proposed method^d^			Reported method [16]
Conc. taken (µg/mL)	Conc. found (µg/mL)	% recovery^a^ ± SD	% recovery^a^ ± SD
1.0	0.99	99.40 ± 1.12	97.70 ± 1.35
3.0	2.96	98.60 ± 0.78	98.320 ± 1.79
10.0	9.95	99.50 ± 1.22	101.02 ± 1.31
14.0	14.22	101.57 ± 1.54	99.63 ± 1.43
16.0	16.05	100.31 ± 0.72	
18.0	17.91	99.50 ± 0.77	
20.0	19.92	99.59 ± 0.34	
${{\bar{\text{X}}}}$ ± SD = 99.78 ± 0.93			99.17 ± 1.47
t-test = 0.86 (2.26)^b^			
*F*-value = 2.5 (8.94)^c^			

^a^Mean recovery of three separate determinations

^b^Tabulated *t-*value at (*P* = 0.05) [27]

^c^Tabulated *F-*value at (*P* = 0.05) [27]

^d^All results of the proposed method were conducted after adjusting excitation wavelength at 451 nm

Repeatability and intermediate precision of the developed spectrofluorimetric method were employed by conducting triplicate assays of aceclofenac using 3 different concentrations (10.0, 14.0, 18.0 µg/mL) in one day and in three successive days, respectively. Small values of the relative standard deviations (RSD) ensured the high precision of the developed spectrofluorimetric method (Table 4).

**Table 4:** Precision data obtained via spectrofluorometric determination of aceclofenac utilizing acriflavine (ACF) as a fluorescent sensor

_Parameters_		Aceclofenac concentration (µg/mL)		
		10.0	14.0	18.0
Intra-day	% Recovery^a^	99.50	102.49	98.09
		101.17	101.57	98.26
		98.80	99.48	99.50
	Mean recovery ± RSD	99.82 ± 1.22	101.18 ± 1.53	99.62 ± 0.78
	% Error	0.70	0.88	0.45
Inter-day	% Recovery^a^	98.31	101.57	99.50
		99.50	102.96	97.98
		101.29	99.62	100.87
	Mean recovery ± RSD	99.70 ± 1.50	101.38 ± 1.66	99.45 ± 1.45
	% Error	0.87	0.96	0.84

^a^Mean value of three individual determinations

All measurements were performed after excitation at 451 nm

The specificity of the developed method was confirmed when the method was applied to analyse aceclofenac in its pharmaceutical preparation (Bristaflam^®^ tablets) where no interferences from the tablets’ excipients were observed.

The robustness of the developed method was confirmed by assessing the influence of small deliberate variation in method parameters on the analytical performance of the developed sensor. These parameters involved pH of the buffer, volume of the buffer, and the volume of reagent. The results indicated that these small changes have no significant effect on the performance of the proposed methods as illustrated in Additional file 1: Table S1.

### Application

The developed fluorescence-based spectroscopic method was utilized to assay aceclofenac in its tablet dosage form. The results were statistically compared with the reported spectrophotometric method [16] by employing student’s *t*-test and variance ratio F-test [27]. The relevance and agreement of the results ensured the aptness of application of the developed method in quality control of aceclofenac in its tablets as demonstrated in Table 5.

**Table 5:** Comparison between the proposed spectrofluorometric method and the reported spectrophotometric for the analysis of aceclofenac in tablet dosage form

Drug (aceclofenac)	Proposed fluorimetric method^e^			Comparison method [16]
	Conc. taken (µg/mL)	Conc. found (µg/mL)	% Recovery^b^	% Recovery^b^
Bristaflam^®^ tablet^a^	10.0	9.80	98.01	98.29
	14.0	14.09	100.62	101.33
	16.0	15.76	98.50	99.59
${{\bar{\text{X}}}}$ ± SD	99.04 ± 1.38			98.94 ± 1.53
*t-*test	0.58 (2.78)^c^			
F-value	1.21 (19.0)^d^			

^a^Bristaflam^®^ labeled to contain 100 mg of aceclofenac in each tablet

^b^Mean recovery of three separate determinations

^c^Tabulated *t*-value at (*P* = 0.05) [25]

^d^Tabulated *F*-value at (*P* = 0.05) [25]

^e^All results of the proposed method were conducted after adjusting excitation wavelength at 451 nm

### Greenness appraisal

The greenness of the developed method was performed using Green Analytical Procedure Index (GAPI) and Analytical GREEnness (AGREE) approaches.

**Fig. 10:**
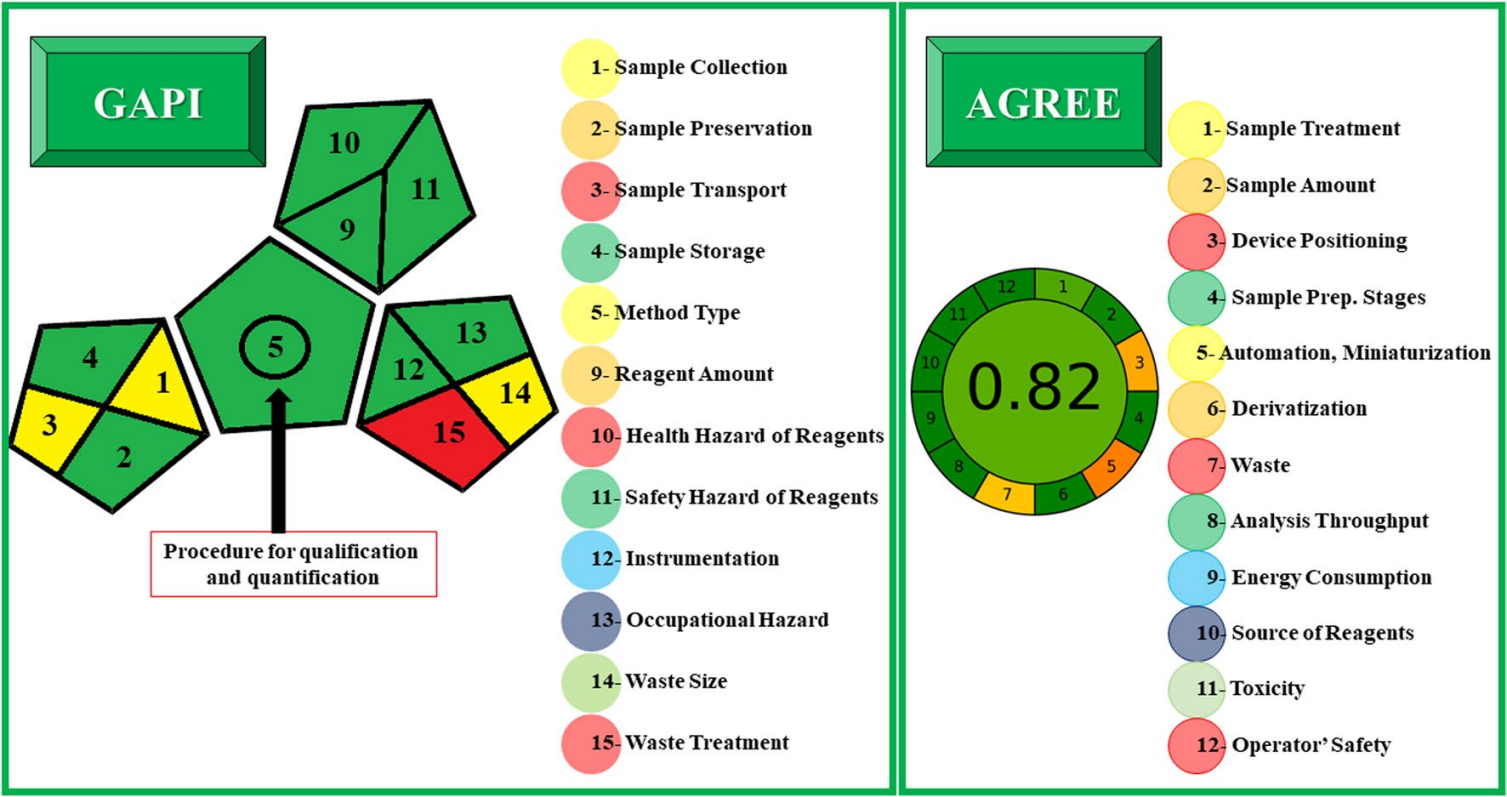
GAPI and AGREE approaches for greenness appraisal of the developed method

GAPI is a pioneering technique that could assess the greenness of the evolved method with performed analytical procedure dependent on fifteen features from sample collection to sample analysis. In accordance with the degree of environmental effect on various parameters, five pentagrams were drawn and colored (each pictogram is divided into 3 or 4 sections) using the GAPI approach, as shown in Fig. 10. A high environmental impact is guaranteed by the color red, a medium environmental effect is conveyed by yellow, and a low environmental impact is indicated by green [28]. The method became more environmentally friendly and sustainable as the number of green-shaded areas increased. Seven green sections were displayed in Fig. 10. This manifestation affirmed the sustainability of the suggested method and supported its suitability for utilization in routine pharmaceutical analysis.

AGREE is an innovative way to assess how environmentally friendly the suggested technique is. The evaluation of AGREE was conducted using the 12 Green Analytical Chemistry (GAC) principles. Twelve portions, each representing one of different GAC principles, make up the perimeter of the clock-like-AGREE graph. The pictogram's unique color (ranging from dark green (1.0) to red (0.0)) represents each parameter's score [29]. The performance's overall color and computed score is shown in the center of the graph. The suggested strategy has a green core color with score of 0.82, which indicates a minimal ecological impact of the evolved method (Fig. 10).

### Comparison of the proposed method with the previously reported methods for the analysis of aceclofenac

Many criteria were considered when comparing the performance of the ACF as a sensing probe for determination of aceclofenac with the other reported ones. First, the sensitivity factor was investigated; the executed approach was found to be 2–103 times more sensitive than some published methods [13–16, 21, 23]. Despite the fact that other reported methods [17–20, 22] were more sensitive than the evolved method, the ecological part that employed green chemistry in the evolved method gave it a competitive advantage. In contrast to previously published methods, all solvents and reagents utilised in the suggested approach were safe and eco-friendly, causing no negative impacts on the environment. Hazardous, toxic, explosive solvents and reagents were employed in significant quantities in previously published methods [13–20, 22, 23]. Furthermore, time is a crucial consideration when developing a new quantitative method. ACF probe is characterized by its ultra-fast determination, in contrast to these methods [17–20] which are time consuming and need lengthy conditioning, washing, and analysis. Only one spectrofluorometric approach [15] for quantification of aceclofenac was described, but it was less sensitive and had a limited linear range when compared to the evolved method. Additional file 1: Table S2 summaries the comparison between the proposed technique and the other published ones. The findings confirmed the superiority of the proposed method over the previously published methods.

## Conclusion

A selective ACF fluorescent probe was developed for simple, precise, and rapid analysis of aceclofenac drug for the first time. The reaction mechanism between aceclofenac and ACF was demonstrated efficiently which shows 1:1 ion-pair complex formation at pH 8.5. The quenching of ACF by aceclofenac was found to be static because the increase in temperature was associated with a decrease in Stern–Volmer constants. The fluorescence quenching was monitored at 502 nm following an excitation at 265 or 451 nm. In addition, the developed fluorescent method was fully validated and efficiently applied to assay aceclofenac in both raw material and dosage form with low RSD (< 1.5%) and high percent recovery (98–101%). The GAPI and AGREE approaches proved the remarkable degree of greenness of the evolved method. High sensitivity, simplicity, rapidity, and sustainability are the key features of the sensor system. This approach is more consistent than the previous published research and is highly recommended for utilization in quality control laboratories because no prior extraction or sample treatment was accomplished.

### Supplementary Information

**Additional file 1: Table S1.** Robustness data for the spectrofluorimetric analysis of aceclofenac. **Table S2.** Comparison between the performance of the developed method and the other reported methods for determination of aceclofenac.

## Data Availability

The datasets generated and/or analysed during the current study are available from the corresponding author upon a reasonable request.

## Abbreviations

A: Absorbance
ACF: Acriflavine
AGREE: The analytical greenness calculator
b: Slope
BRB: Britton Robinson buffer
C: Concentration
COVID-19: Corona virus disease 2019
λ_ex_: Excitation wavelength
λ_em_: Emission wavelength
F: Fluorescence
Φ: Quantum yield
GAC: Green analytical chemistry
GAPI: Green analytical procedure index
HIV: Human immunodeficiency virus
ICH: International Council on Harmonisation
K_SV_: Stern–Volmer constant
Kq: Bimolecular quenching constant
LC–MS/MS: Liquid chromatography–tandem mass spectrometry
LOD: Limit of detection
LOQ: Limit of quantification
η: Solvent refractive index
r: Correlation coefficient
RFI: Relative fluorescence intensity
RP-HPLC: Reversed-phase high-performance liquid chromatography
RSD: Relative standard deviation
S_a_: Standard deviation of intercept
S_b_: Standard deviation of slope
SD: Standard deviation
st: The standard
t_0_: Luminescence lifetime
x: The unknown sample

## References

[1] Piorecka K, Kurjata J, Stanczyk WA (2022). Acriflavine, an acridine derivative for biomedical application: current state of the art. J Med Chem.

[2] Nehme R, Hallal R, El Dor M, Kobeissy F, Gouilleux F, Mazurier F, Zibara K (2021). Repurposing of acriflavine to target chronic myeloid leukemia treatment. Curr Med Chem.

[3] Dana S, Prusty D, Dhayal D, Gupta MK, Dar A, Sen S, Mukhopadhyay P, Adak T, Dhar SK (2014). Potent antimalarial activity of acriflavine in vitro and in vivo. ACS Chem Biol.

[4] Mathé G, Triana K, Pontiggia P, Blanquet D, Hallard M, Morette C (1998). Data of pre-clinical and early clinical trials of acriflavine and hydroxy-methyl-ellipticine reviewed, enriched by the experience of their use for 18 months to 6 years in combinations with other HIV1 virostatics. Biomed Pharmacother.

[5] Lee K, Zhang H, Qian DZ, Rey S, Liu JO, Semenza GL (2009). Acriflavine inhibits HIF-1 dimerization, tumor growth, and vascularization. Proc Natl Acad Sci USA.

[6] Napolitano V, Dabrowska A, Schorpp K, Mourão A, Barreto-Duran E, Benedyk M, Botwina P, Brandner S, Bostock M, Chykunova Y (2022). Acriflavine, a clinically approved drug, inhibits SARS-CoV-2 and other betacoronaviruses. Cell Chem Biol.

[7] Abd Ali LI, Qader AF, Salih MI, Aboul-Enein HY (2019). Sensitive spectrofluorometric method for the determination of ascorbic acid in pharmaceutical nutritional supplements using acriflavine as a fluorescence reagent. Luminescence.

[8] Tolba M, Elmansi H (2021). Studying the quenching resulted from the formation of an association complex between olsalazine or sulfasalazine with acriflavine. R Soc Open Sci.

[9] Abdel-Aziz H, Tolba MM, El-Enany N, Aly FA, Fathy ME (2021). Green and sensitive spectrofluorimetric method for the determination of two cephalosporins in dosage forms. R Soc Open Sci.

[10] Dagher D, Elmansi H, Nasr JJ, El-Enany N (2022). Utility of a novel turn-off fluorescence probe for the determination of tranilast, an adjunctive drug for patients with severe COVID-19. RSC Adv.

[11] Iolascon G, Giménez S, Mogyorósi D (2021). A review of aceclofenac: analgesic and anti-inflammatory effects on musculoskeletal disorders. J Pain Res.

[12] Legrand E (2004). Aceclofenac in the management of inflammatory pain. Expert Opin Pharmacother.

[13] Aderibigbe SA, Adegoke OA, Idowu OS, Olaleye SO (2012). Sensitive spectrophotometric determination of aceclofenac following azo dye formation with 4-carboxyl-2,6-dinitrobenzene diazonium ion. Acta Pol Pharm.

[14] Bose A, Dash PP, Sahoo MK (2010). Simple spectrophotometric methods for estimation of aceclofenac from bulk and formulations. Pharm Methods.

[15] El Kousy NM (1999). Spectrophotometric and spectrofluorimetric determination of etodolac and aceclofenac. J Pharm Biomed Anal.

[16] Saravanan VS, Ware A, Natesan G (2006). UV-spectrophotometric determination of aceclofenac in tablets. Asian J Chem.

[17] Kang W, Kim E-Y (2008). Simultaneous determination of aceclofenac and its three metabolites in plasma using liquid chromatography–tandem mass spectrometry. J Pharm Biomed Anal.

[18] Ojha A, Rathod R, Padh H (2009). Simultaneous HPLC–UV determination of rhein and aceclofenac in human plasma. J Chromatogr B.

[19] Kim E, Ahn B, Noh K, Kang W, Gwak H (2012). Quantitative determination of aceclofenac and its three major metabolites in rat plasma by HPLC-MS/MS. J Sep Sci.

[20] Lee HS, Jeong CK, Choi SJ, Kim SB, Lee MH, Ko GI, Sohn DH (2000). Simultaneous determination of aceclofenac and diclofenac in human plasma by narrowbore HPLC using column-switching. J Pharm Biomed Anal.

[21] Zinellu A, Carru C, Sotgia S, Porqueddu E, Enrico P, Deiana L (2005). Separation of aceclofenac and diclofenac in human plasma by free zone capillary electrophoresis using N-methyl-d-glucamine as an effective electrolyte additive. Eur J Pharm Sci.

[22] Górska A, Paczosa-Bator B, Gaidukevič J, Piech R (2022). Development of a new voltammetric method for aceclofenac determination on glassy carbon electrode modified with hierarchical nanocomposite. Sensors.

[23] Posac JR, Vázquez MD, Tascón ML, Acuña JA, de la Fuente C, Velasco E, Sánchez-Batanero P (1995). Determination of aceclofenac using adsorptive stripping voltammetric techniques on conventional and surfactant chemically modified carbon paste electrodes. Talanta.

[24] Würth C, Grabolle M, Pauli J, Spieles M, Resch-Genger U (2013). Relative and absolute determination of fluorescence quantum yields of transparent samples. Nat Protoc.

[25] Magde D, Wong R, Seybold PG (2002). Fluorescence quantum yields and their relation to lifetimes of rhodamine 6G and fluorescein in nine solvents: improved absolute standards for quantum yields. Photochem Photobiol.

[26] ICH Harmonized Tripartite Guideline, Validation of Analytical Procedures: Text and Methodology, Q2(R1), Current Step 4 Version, Parent Guidelines on Methodology Dated November 6 1996, Incorporated in November 2005. .http://www.ich.org/products/guidelines/quality/article/quality-guidelines.html.

[27] Miller JN, Miller JC (2010). Statistics and chemometrics for analytical chemistry.

[28] Płotka-Wasylka J (2018). A new tool for the evaluation of the analytical procedure: Green Analytical Procedure Index. Talanta.

[29] Pena-Pereira F, Wojnowski W, Tobiszewski M (2020). AGREE—analytical GREEnness metric approach and software. Anal Chem.

