# Supplementary material for: Acriflavine: an efficient green fluorescent probe for sensitive analysis of aceclofenac in pharmaceutical formulations
Source: BMC Chem. 2023 Aug 2;17(1):93. doi: 10.1186/s13065-023-00979-2 (PMC10394782; doi:10.1186/s13065-023-00979-2)
Supplement: Supplementary file 1 — Additional file 1: Table S1. Robustness data for the spectrofluorimetric analysis of aceclofenac. Table S2. Comparison between the performance of the developed method and the other reported methods for determination of aceclofenac. [file 13065_2023_979_MOESM1_ESM.docx]

**Acriflavine: An Efficient Green Fluorescent Probe for Sensitive Analysis of Aceclofenac in Pharmaceutical Formulations**

**Amal A. El-Masry^a^ and Abdallah M. Zeid^b*^**

^a^Department of Medicinal Chemistry, Faculty of Pharmacy, Mansoura University, 35516 Mansoura, Egypt

^b^Department of Pharmaceutical Analytical Chemistry, Faculty of Pharmacy, Mansoura University, 35516 Mansoura, Egypt

*Corresponding author email address: dr_abdallah_zeid@mans.edu.eg

**Additional Information**

**Table S1: Robustness data for the spectrofluorimetric analysis of aceclofenac.**

| **Parameters** | **Volume of buffer solution (mL)** | | | **pH of buffer solution** | | | **Volume of acriflavine (mL)** | | |
| --- | --- | --- | --- | --- | --- | --- | --- | --- | --- |
|  | **0.8** | **1.0** | **1.2** | **8.3** | **8.5** | **8.7** | **0.98** | **1.0** | **1.02** |
| **% Recovery** | 99.94 | 99.50 | 99.31 | 98.99 | 99.50 | 99.23 | 99.65 | 99.50 | 100.03 |
| **$\bar{\text{X}}$± SD** | 99.58 ± 0.32 | | | 99.24 ± 0.26 | | | 99.73 ± 0.27 | | |
| **% Error** | 0.19 | | | 0.15 | | | 0.16 | | |

| **Table S2: Comparison between the performance of the developed method and the other reported methods for determination of aceclofenac.** | | | | | |
| --- | --- | --- | --- | --- | --- |
| **Methods** | **Conditions** | **Linearity range (μg/mL)** | **LOD (μg/mL)** | **Solvent** | **Ref.** |
| Spectrophotometry | Spectrophotometric determination following azo dye formation with 4-carboxyl-2,6-dinitrobenzenediazonium ion. Measurement of the absorbance was carried out at 430 nm. | 1.2 - 4.8 | 0.4 | Glacial acetic acid | [1] |
|  | Determination of aceclofenac after its reaction with either (p-dimethylaminocinnamaldehyde or 3-Methyl-2-benzothiazolinone hydrazine hydrochloride and measuring the chromogen at the λ_max_ by 658 and 592, respectively. | 1.0 – 100.0 | 0.91 | Methanol | [2] |
|  | Formation of colored complexes between the drugs and p-dimethylaminobenzaldehyde reagent in the presence of sulfuric acid and ferric chloride. Measurement of the absorbance was carried out at 545.5 nm. | 8.0 – 55.0 | - | Ethanol | [3] |
|  | Direct spectrophotometric determination of aceclofenac. Measurement of the absorbance was carried out at 203 nm. | 2.0 – 10.0 | - | Methanol: Water (50: 50) | [4] |
| Spectrofluorimetry | Direct determination of the fluorescence at wavelength 355 nm after excitation at wavelength 250 nm. | 2.0 - 8.0 | - | Phosphate buffer  (pH 8.0) | [3] |
|  | Using acriflavine as an efficient fluorescent probe. The fluorescence quenching was monitored at 502 nm following an excitation at 265 or 451 nm. | 1.0 - 20.0 | 0.29 | Water | This method |
| LC-MS/MS | C_18_ column, mobile phase consisted of acetonitrile: 0.1% formic acid (80: 20, v/v). | 0.1 - 20.0 | 0.002 | Acetonitrile | [5] |
| RP-HPLC | C_18_ column, mobile phase consisted of ammonium acetate buffer (0.01 M; pH 5.65) and acetonitrile (74: 26, v/v). | 0.5 – 20.0 | - | Methanol | [6] |
| LC-MS/MS | C_18_ column, mobile phase consisted of acetonitrile: 0.1% formic acid (9: 1; v/v) | 0.01 – 50.0 | 0.002 | Methanol | [7] |
| RP-HPLC | Phenyl hexyl column, mobile phase consisted of acetonitrile: 0.02 M potassium phosphate (33: 67; v/v, pH 7.0). | 0.05 – 10 .0 | 0.01 | Methanol | [8] |
| Capillary electrophoresis | 40 cm x 75 microm uncoated silica capillary, background electrolyte consisted of 300.0 mmol/L sodium borate buffer and 200.0 mmol/L N-methyl-D-glucamine, pH 8.9. | 2500 - 40000 | 30.0 | 300.0 mmol/L sodium borate and 200.0 mmol/L N-methyl-D-glucamine, pH 8.9. | [9] |
| Electrochemichal | Voltammetric method; a glassy carbon electrode (GCE) modified with carbon nanofibers, carbon nanotubes, and NiCo nanoparticles (eCNF/CNT/NiCo-GCE) was used. | 0.02 - 0.5 | 0.0003 | Dimethyl sulfoxide  (DMSO) | [10] |
|  | Adsorptive stripping voltammetric method; a conventional and surfactant chemically modified electrode was used. | - | 7.0 | Ethanol | [11] |

**References:**

1. Aderibigbe SA, Adegoke OA, Idowu OS, Olaleye SO: **Sensitive spectrophotometric determination of aceclofenac following azo dye formation with 4-carboxyl-2,6-dinitrobenzene diazonium ion**. *Acta poloniae pharmaceutica* 2012, **69**(2):203-211.

2. Bose A, Dash PP, Sahoo MK: **Simple spectrophotometric methods for estimation of aceclofenac from bulk and formulations**. *Pharmaceutical Methods* 2010, **1**(1):57-60.

3. El Kousy NM: **Spectrophotometric and spectrofluorimetric determination of etodolac and aceclofenac**. *J Pharm Biomed Anal* 1999, **20**(1):185-194.

4. Saravanan VS, Ware A, Natesan G: **UV-spectrophotometric determination of aceclofenac in tablets**. *Asian J Chem* 2006, **18**:3251-3252.

5. Kang W, Kim E-Y: **Simultaneous determination of aceclofenac and its three metabolites in plasma using liquid chromatography–tandem mass spectrometry**. *J Pharm Biomed Anal* 2008, **46**(3):587-591.

6. Ojha A, Rathod R, Padh H: **Simultaneous HPLC–UV determination of rhein and aceclofenac in human plasma**. *J Chromatogr B* 2009, **877**(11):1145-1148.

7. Kim E, Ahn B, Noh K, Kang W, Gwak H: **Quantitative determination of aceclofenac and its three major metabolites in rat plasma by HPLC-MS/MS**. *J Sep Sci* 2012, **35**(17):2219-2222.

8. Lee HS, Jeong CK, Choi SJ, Kim SB, Lee MH, Ko GI, Sohn DH: **Simultaneous determination of aceclofenac and diclofenac in human plasma by narrowbore HPLC using column-switching**. *J Pharm Biomed Anal* 2000, **23**(5):775-781.

9. Zinellu A, Carru C, Sotgia S, Porqueddu E, Enrico P, Deiana L: **Separation of aceclofenac and diclofenac in human plasma by free zone capillary electrophoresis using N-methyl-d-glucamine as an effective electrolyte additive**. *Eur J Pharm Sci* 2005, **24**(4):375-380.

10. Górska A, Paczosa-Bator B, Gaidukevič J, Piech R: **Development of a New Voltammetric Method for Aceclofenac Determination on Glassy Carbon Electrode Modified with Hierarchical Nanocomposite**. *Sensors (Basel, Switzerland)* 2022, **22**(22).

11. Posac JR, Vázquez MD, Tascón ML, Acuña JA, de la Fuente C, Velasco E, Sánchez-Batanero P: **Determination of aceclofenac using adsorptive stripping voltammetric techniques on conventional and surfactant chemically modified carbon paste electrodes**. *Talanta* 1995, **42**(2):293-304.
